# PFOS Disturbs BDNF-ERK-CREB Signalling in Association with Increased MicroRNA-22 in SH-SY5Y Cells

**DOI:** 10.1155/2015/302653

**Published:** 2015-11-15

**Authors:** Wu Li, Qing-zhi He, Cheng-qiu Wu, Xiao-yuan Pan, Jing Wang, Yan Tan, Xiao-yun Shan, Huai-cai Zeng

**Affiliations:** ^1^Department of Preventive Medicine, School of Public Health, University of South China, 28 Western Changsheng Road, Hengyang 421001, China; ^2^Hunan Province Cooperative Innovation Center for Molecular Target New Drug Study, 28 Western Changsheng Road, Hengyang 421001, China

## Abstract

Perfluorooctane sulfonate (PFOS), a ubiquitous environmental pollutant, is neurotoxic to mammalian species. However, the underlying mechanism of its neurotoxicity was unclear. We hypothesized that PFOS suppresses BDNF expression to produce its neurotoxic effects by inhibiting the ERK-CREB pathway. SH-SY5Y human neuroblastoma cells were exposed to various concentrations of PFOS to examine the role of the BDNF-ERK-CREB signalling pathway in PFOS-induced apoptosis and cytotoxicity. Furthermore, to ascertain the mechanism by which PFOS reduces BDNF signalling, we examined the expression levels of miR-16 and miR-22, which potentially regulate BDNF mRNA translation at the posttranscriptional level. Results indicated that PFOS significantly decreased cell viability and induced apoptosis in SH-SY5Y cells. In addition, BDNF and pERK protein levels decreased after PFOS treatment; however, pCREB protein levels were significantly elevated in PFOS treated groups. TrkB protein expression increased in the 10 *μ*M and 50 *μ*M PFOS groups and significantly decreased in the 100 *μ*M PFOS group. Our results demonstrated that PFOS exposure decreased miR-16 expression and increased miR-22 expression, which may represent a possible mechanism by which PFOS decreases BDNF protein levels. PFOS may inhibit BDNF-ERK-CREB signalling by increasing miR-22 levels, which may, in part, explain the mechanism of PFOS neurotoxicity.

## 1. Introduction

Perfluorooctane sulfonate (PFOS), a persistent organic pollutant and the final degradation product of many perfluorinated compounds [1, 2], is extensively used in industrial, commercial, and household applications due to its exceptional hydrophobic and oleophobic properties; PFOS is found in carpets, textiles, leather, paper, fire-fighting foams, food packing materials, and components of pharmaceuticals and insecticides [3]. PFOS exists in water, air, and environmental media sediments and is also found in animals, including marine mammals, fish, birds [4], and humans [5]. The median serum PFOS levels in residents and occupationally exposed people in China were reported as 34.16 ng/mL and 33.46 ng/mL, respectively [6]. PFOS strongly binds to plasma albumin; its long half-life in human serum (5.4 y) [7] is 20 times that in rats [8]. In 2002, when the 3M Company ceased quantifying PFOS production, the total worldwide production of PFOS was estimated to be 122,500 tons (including unusable wastes from 1970 to 2002) [9]. In mammals, PFOS predominantly accumulates in the liver and serum as well as in the brain [10]. PFOS has been shown to cause various adverse health effects, including hepatotoxicity [11], developmental toxicity [8], cardiotoxicity [12], immunotoxicity [13], reproductive toxicity [11], and neurotoxicity [14]. PFOS is capable of crossing the blood-brain [15] and blood-placental barriers [16], and it was detected in human breast milk samples at a mean concentration of 0.201 ng/mL [5]; thus, developing foetal and infant brains may be vulnerable to PFOS exposure. Previous studies revealed that PFOS exposure induced an inflammation-like glial response in the developing rat brain [17], caused neurobehavioral defects [18], and impaired spatial learning and memory in mice [19, 20]. However, the mechanism of PFOS-induced neurotoxicity has not been fully elucidated.

Brain-derived neurotrophic factor (BDNF) is a critical neurotrophic factor that influences neuronal proliferation, synaptic function, and synaptic plasticity via binding to tropomyosin-related kinase B (TrkB). TrkB binding promotes the activation of intracellular signalling cascades, including the mitogen-activated protein kinase/extracellular signal-regulated kinase (MAPK/ERK), phospholipase C*γ* (PLC*γ*), and phosphatidylinositol 3-kinase (PI3K) pathways [21, 22]. Phosphorylated ERK promotes cAMP-response element binding protein (CREB) phosphorylation indirectly through its downstream signalling molecules, RSKs (ribosomal S6 kinases), and MSKs (mitogen and stress activated kinases). BDNF transcription is then promoted when phosphorylated CREB (pCREB) binds to the BDNF promoter region [23]. The BDNF-ERK-CREB pathway plays a critical role in various neuronal biological processes, including cell survival, synaptic structure, and synaptic plasticity [24, 25].

MicroRNAs (miRNAs) are evolutionarily conserved, small, noncoding RNAs that posttranscriptionally inhibit protein coding genes by affecting translation and/or mRNA stability [26]. The effect of particular miRNAs on the posttranscriptional control of BDNF expression is beginning to be elucidated [27]. miRNA-16 and miRNA-22 are two such mediators that are known to inhibit BDNF expression [28, 29]. Therefore, we hypothesized that they may be involved in PFOS-induced neurotoxicity.

The human neuroblastoma cell line SH-SY5Y is a thrice cloned subline of the neuroblastoma cell line SK-N-SH (SK-N-SH -> SH-SY -> SH-SY5 -> SH-SY5Y) and is widely used to determine the neurotoxicity of xenobiotics. To better understand the potential molecular mechanism of neurotoxicity induced by PFOS, we evaluated the general cytotoxicity of PFOS in SH-SY5Y cells and investigated the effects of PFOS on the BDNF-ERK-CREB pathway. Additionally, we explored the mechanism by which PFOS affected BDNF expression via miRNA regulation.

## 2. Materials and Methods

### 2.1. Chemicals and Reagents

Perfluorooctane sulfonic acid potassium salt (PFOS, ≥98% purity) and dimethyl sulfoxide (DMSO) were purchased from Fluka (Sigma-Aldrich, Switzerland). PFOS was dissolved in DMSO at a maximum concentration of 200 mM, stored at −20°C, and diluted to the desired concentrations in culture medium immediately before use. The final concentration of DMSO in the culture medium did not exceed 0.1%. All chemicals used in this study were commercially available and of appropriate grades.

### 2.2. Cell Culture and PFOS Treatment

SH-SY5Y cells (kindly provided by the Institute of Animal Studies, Beijing, China) were grown in DMEM (HyClone, USA) supplemented with 10% FBS (HyClone, USA), 100 U/mL penicillin, and 100 *μ*g/mL streptomycin at 37°C in 5% CO_2_. Cells were passaged every 2 to 3 days as follows: media with floating cells was collected, and the adherent cells were washed with PBS (pH = 7.4), dissociated with 1 to 2 mL of 0.125% trypsin solution (BIOSHARP, China) at room temperature or 37°C, and recovered by centrifugation at 1000 rpm for 6 min. Fresh medium was added, and the cells were aspirated, combined with the floating cells recovered above, and dispensed into new flasks. Cells were seeded on flat bottom plates at 5000 cells/cm^2^, maintained in a humidified incubator with 5% CO_2_ at 37°C for 24 h until they adhered completely, and then incubated with PFOS solutions for further assays while in the exponential growth phase.

### 2.3. Cell Viability Assay and Morphological Study

Cell viability was determined by CCK8 assay. After a 24- or 48-hour exposure to PFOS (1, 10, 50, 100, 150, and 200 *μ*M) or control medium (0.1% DMSO), cells in 96-well plates were incubated in 10% CCK-8 (Dojindo, Japan) diluted in normal culture medium at 37°C until colour conversion could be visualized. Each dose group was analysed in five replicate wells to reduce intragroup deviation. After a 1- to 4-hour incubation, the absorbance was measured at 450 nm using enzyme-linked immunosorbent assay plate reader (BIO-TEK ELX-800, USA). The morphology of SH-SY5Y cells on 12-well plates was examined and recorded using an inverted microscope (Olympus CKX41, Japan) within one hour of the 48-hour exposure to PFOS (10, 50, 100, 150, and 200 *μ*M) or control medium (0.1% DMSO).

### 2.4. Apoptosis Evaluation by Dual AO/EB Fluorescent Staining

Cell apoptosis was evaluated using an AO (acridine orange, 100 *μ*g/mL)/EB (ethidium bromide, 100 *μ*g/mL) fluorescence staining assay (AO/EB, Sigma, USA). SH-SY5Y cells seeded in 12-well plates were exposed to PFOS (10, 50, 100, 150, and 200 *μ*M) or control medium (0.1% DMSO) for 48 h, and then the cells were stained with AO/EB solution (1 : 1 v/v) for 10 min. The changes in SH-SY5Y cell nuclear morphology were immediately visualized and recorded using an inverted fluorescent microscope (Leica DM IL, German).

### 2.5. RNA Extraction and cDNA Synthesis

Total RNA was isolated using TRIZOL (Invitrogen, USA) according to the manufacturer's instructions. The ratio of the optical densities of the RNA samples at 260 and 280 nm was used to evaluate nucleic acid purity, and total RNA concentrations were determined based on the absorbance at 260 nm. The total RNA quality was further estimated based on the integrity of 28S and 18S RNA after 1% agarose gel electrophoresis. cDNA synthesis for coding genes and miRNAs was performed with 1 *μ*g of total RNA using the Thermo Scientific RevertAid First Strand cDNA Synthesis Kit (Thermo, USA) and miScript II RT Kit (Qiagen, USA), respectively, according to the manufacturers' instructions. cDNA was stored at −80°C.

### 2.6. Real-Time Quantitative PCR (QPCR)

mRNA expression levels (BDNF, CREB, ERK1, ERK2, and *β*-Actin) were analysed using SYBR Green Supermix (Bio-Rad, USA) according to the manufacturer's instructions. The identity of the analysed coding genes and their corresponding forward and reverse primer sequences are listed in Table 1. Gene-specific primers were synthesised by Guangzhou Invitrogen Corporation. The conditions for qRT-PCR were as follows: 95°C for 10 min followed by 40 cycles of 95°C for 15 s (denaturation), 55°C for 30 s (annealing), and 72°C for 30 s (extension) on an iQ5 Multicolor Real-Time PCR Detection System (Bio-Rad, USA). The expression levels of specific miRNAs (has-miR-16 and has-miR-22-3p) were analysed using the miScript SYBR Green PCR Kit and miScript Primer Assays (Qiagen, USA) according to the manufacturer's instructions and normalized to U6 expression; the corresponding primer sequences used for miRNA reverse transcription and QPCR were not listed in the instructions. Each sample was run in triplicate for the target and reference genes, along with a single NRT (no reverse transcriptase) negative control, in a total reaction volume of 25 *μ*L. *β*-Actin and U6, which were uniformly expressed across all samples (cycle threshold standard deviation less than 0.5), were used as endogenous reference genes. Dissociation curve analysis was performed for each gene to check for amplification of untargeted fragments. Only one peak was observed for each reaction, indicating the amplification of only the target gene. Fold changes in target gene expression were calculated using the 2^−ΔΔCT^ (CT, cycle threshold) method.

### 2.7. Enzyme-Linked Immunosorbent Assay (ELISA)

The protein levels of BDNF were determined using a Human BDNF ELISA Kit (RayBio, England) according to the manufacturer's instructions. After a 48-hour exposure to PFOS (10, 50, and 100 *μ*M) or control medium (0.1% DMSO), the supernatants of SH-SY5Y cells cultured in 6-well plates were collected and centrifuged at 1000 rpm at 4°C to remove floating cells, and then the prepared supernatants were immediately added to a 96-well plate coated with a primary antibody specific to human BDNF. The wells were washed, and the secondary biotinylated anti-human BDNF antibody was added, followed by the addition of HRP-conjugated streptavidin. TMB substrate solution was added to the wells as the developing agent, and colour developed in proportion to the amount of bound BDNF. Finally, the Stop Solution changed the colour from blue to yellow, and the intensity was measured at 450 nm. A standard curve was run for each assay, and all standards or samples were run in triplicate.

### 2.8. Western Blot Analysis

Total cellular proteins were extracted using RIPA lysis buffer (Beyotime, China) containing protease (1 mM PMSF, Beyotime, China) and phosphatase (10 nM NaF, Beyotime, China) inhibitors at 4°C. Protein concentrations were determined using a bicinchoninic acid (BCA) protein assay kit (Beyotime, China). Equal amounts of protein (100 *μ*g) from each sample were solubilized in sample buffer (25 mM Tris, PH 6.8, 1% SDS (w/v), 5% *β*-mercaptoethanol (v/v), 1 mM EDTA, 4% glycerol, and 0.01% bromophenol blue) and separated by 12% SDS-polyacrylamide gel electrophoresis. The proteins were then transferred to a nitrocellulose membrane. Nonspecific binding was reduced by incubating the membrane in a blocking buffer (Beyotime, Shanghai, China) for 1 h. The membrane with transferred protein was incubated in buffer containing specific primary antibodies (anti-TrkB (SANTA, 1 : 1000), anti-CREB (SANTA, 1 : 1000), anti-pCREB (SANTA, 1 : 1000), anti-ERK1/2 (Abcam, 1 : 1000), anti-pERK1/2 (Abcam, 1 : 1000), and anti-*β*-Actin (Abcam, 1 : 1000)) overnight at 4°C, washed three times with TBST for 15 min, and incubated with horseradish peroxidase-conjugated goat anti-rabbit IgG (1 : 1000; Beyotime, Shanghai, China) or horseradish peroxidase-conjugated goat anti-mouse IgG (1 : 1000; Beyotime, Shanghai, China) for 1 h. Specific signals were detected using BeyoECL Plus (Beyotime, Shanghai, China). The amount of *β*-Actin (42 kDa) in each lane was used as an internal control for the amount of TrkB (92 kDa), CREB (43 kDa), pCREB (43 kDa), ERK1/2 (44/42 kDa), and pERK1/2 (p44/42 kDa).

### 2.9. Statistical Analysis

All data are presented as mean ± SD, and statistical analyses were performed with one-way ANOVA followed by LSD and Dunnett's T3 test using SPSS 17.0. At the same time, we used a two-way ANOVA as statistical analysis method to analyse the effects of PFOS concentration and exposure time on SH-SY5Y cell viability. A *p* value < 0.05 was considered statistically significant in all experiments.

## 3. Results

### 3.1. Effects of PFOS on SH-SY5Y Cell Viability and Morphology

**Figure 1:**
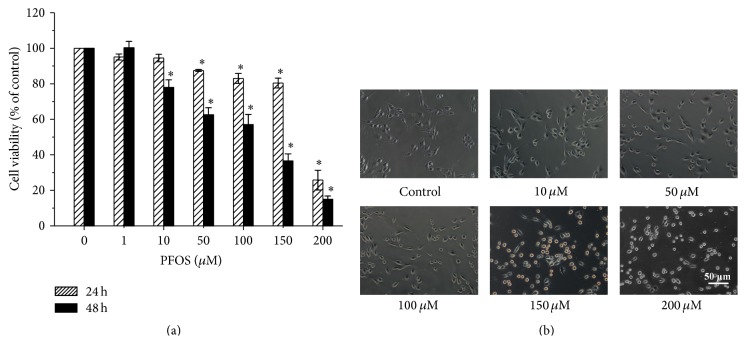
Effects of PFOS on SH-SY5Y cell viability and morphology. (a) Cell viability was determined by CCK8 assay after a 24- or 48-hour exposure to various concentrations of PFOS (1, 10, 50, 100, 150, and 200 *μ*M) or DMSO (control). (b) SH-SY5Y cell morphology was observed after a 48-hour exposure to various concentrations of PFOS (10, 50, 100, 150, and 200 *μ*M) or DMSO (control). Magnification, 200x. Data are presented as mean ± SD of three separate experiments. ^*∗*^ *p* < 0.05, experimental values compared with control values.

To identify the effects of PFOS on cell viability and morphology, SH-SY5Y cells were exposed to various concentrations of PFOS or DMSO (control) for 24 or 48 h. As shown in Figure 1(a), PFOS significantly decreased cell viability at 50 *μ*M at 24 h and at 10 *μ*M at 48 h. At the same time, a two-way ANOVA analysis indicated that both of PFOS exposure concentrations (*F* = 10.69, *p* = 0.005 < 0.05) and PFOS exposure time (*F* = 6.96, *p* = 0.039 < 0.05) decreased cell viability significantly, which means, besides PFOS exposure concentration, PFOS exposure time was also a factor which influenced cell viability. We used 48 h as the detection point for further analyses as it represents the population doubling time of SH-SY5Y cells. After a 48-hour incubation with 10 *μ*M PFOS, the morphology of SH-SY5Y cells was similar to that of control DMSO-treated cells (Figure 1(b)). However, increasing the concentration of PFOS to 50 *μ*M resulted in the shrinking of cell bodies, the obscuring of cellular boundaries, and the disappearance of neurites as well as an increase in the number of cells with irregular morphology and of dead cells suspended in the culture solution. Because SH-SY5Y cell viability was less than 60% after treatment with 100 *μ*M PFOS, concentrations less than or equal to 100 *μ*M were used in the subsequent studies.

### 3.2. PFOS Induced Apoptosis in SH-SY5Y Cells

**Figure 2:**
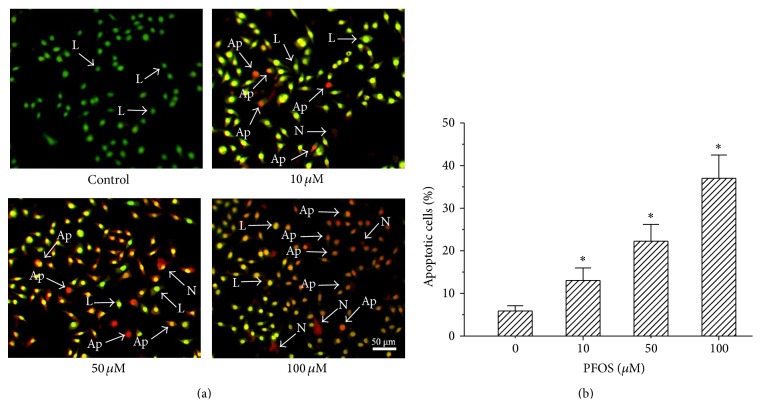
Effects of PFOS on the apoptosis of SH-SY5Y cells. (a and b) Cells were treated with DMSO (vehicle control) or PFOS (10, 50, and 100 *μ*M) for 48 h. Nuclear morphology was monitored by fluorescent inverted microscopy after staining the cells with AO/EB fluorescent dyes. Magnification, 100x. Six hundred cells were analysed, and the number of apoptotic cells was expressed as a percentage of the total number of analysed cells. L: live cells; Ap: apoptotic cells; N: necrotic cells. Data are presented as mean ± SD of three independent experiments. ^*∗*^ *p* < 0.05, experimental values compared with control values.

Apoptotic cell death after exposure to PFOS was determined by evaluating nuclear morphology using dual AO/BR fluorescent staining. Treating cells with 10, 50, or 100 *μ*M PFOS for 48 h induced apparent chromatin or nuclear condensation and fragmentation, typical consequences of apoptosis (Figure 1(a)). The percentage of apoptotic cells increased by 13.06% and 37.02% at 10 and 100 *μ*M PFOS, respectively (Figure 2(b)), indicating significant PFOS-induced apoptosis.

### 3.3. Effects of PFOS on the BDNF-TrkB-CREB Signalling Pathway in SH-SY5Y Cells

**Figure 3:**
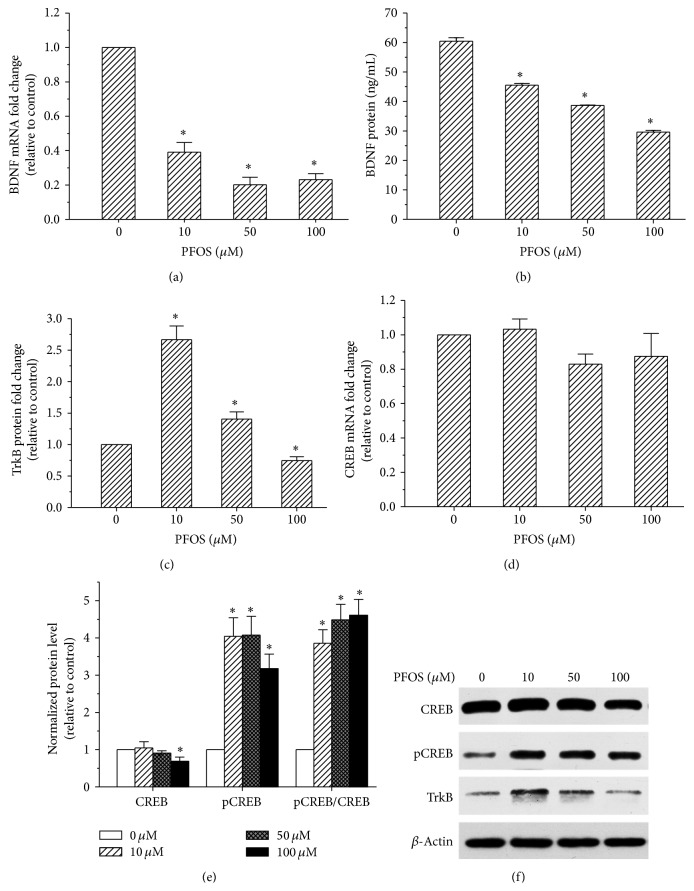
Effects of PFOS on the expression of BDNF, TrkB, and CREB in SH-SY5Y cells. Cells were cultured for 48 h with various concentrations of PFOS (10, 50, and 100 *μ*M) or DMSO (control). (a) BDNF mRNA levels in SH-SY5Y cells. (b) BDNF protein levels in the culture supernatant from SH-SY5Y cells. (c) TrkB protein levels in SH-SY5Y cells. (d) CREB mRNA levels in SH-SY5Y cells. (e) CREB and pCREB protein levels and the pCREB/CREB ratio in SH-SY5Y cells; the band intensities of pCREB and CREB were quantified, and the fold increases in the ratio of pCREB/CREB are presented as a bar graph. (f) The protein levels of TrkB, CREB, and pCREB were determined by Western blot analysis using *β*-Actin as an internal control. Each data point was normalized to the control (DMSO), and the data are presented as mean ± SD from three independent experiments. ^*∗*^ *p* < 0.05, experimental values compared with control values.

The expression levels of BDNF, TrkB, and CREB were measured to determine the effect of PFOS on the BDNF-TrkB-CREB signalling pathway. Compared with controls, the mRNA and protein levels of BDNF were both significantly reduced after a 48-hour incubation with 10, 50, or 100 *μ*M PFOS (Figures 3(a) and 3(b)).

Our results indicated that CREB mRNA and protein levels were unaffected by PFOS treatment, except at 100 *μ*M, at which CREB protein levels decreased compared with control treatment. However, compared with the controls, CREB phosphorylation increased to 404%, 407%, and 317% and the pCREB/CREB ratio increased to 385%, 448%, and 460% in the 10, 50, and 100 *μ*M PFOS treatment groups, respectively, after a 48-hour incubation (Figures 3(d), 3(e), and 3(f)). Furthermore, compared with the control, TrkB protein levels significantly increased in the 10 and 50 *μ*M dose groups. Notably, TrkB protein levels significantly decreased in the 100 *μ*M dose group compared with the control group (Figure 3(c)). These results indicate that PFOS interfered with the BDNF-TrkB-CREB signalling pathway in SH-SY5Y cells.

### 3.4. Effect of PFOS on the ERK1/2 Pathway in SH-SY5Y Cells

**Figure 4:**
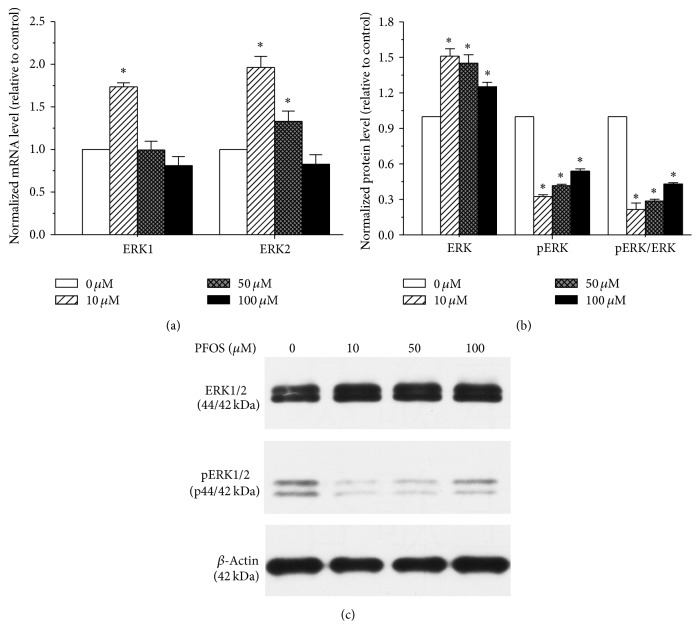
Effects of PFOS on ERK activation in SH-SY5Y cells. (a) ERK1 and ERK2 mRNA levels in SH-SY5Y cells. (b) ERK and pERK protein levels and the pERK/ERK ratio in SH-SY5Y cells; the band intensities of pERK and ERK were quantified, and the fold increases in the ratio of pERK/ERK are presented as a bar graph. (c) The protein levels of ERK1/2 and pERK1/2 were determined by Western blot analysis using *β*-Actin as an internal control. Each data point was normalized to the control (DMSO), and the data are presented as mean ± SD from three independent experiments. ^*∗*^ *p* < 0.05, experimental values compared with control values.

The expression levels of ERK1 and ERK2 and ERK phosphorylation were measured by qPCR and Western blotting to determine the effects of PFOS on the ERK1/2 pathway. As shown in Figure 4, compared with the control, ERK1 mRNA expression increased significantly in the 10 *μ*M group only; ERK2 mRNA expression increased significantly in the 10 *μ*M and 50 *μ*M dose groups. ERK protein levels increased by 150%, 145%, and 125% in the 10, 50, and 100 *μ*M dose groups, respectively. However, ERK phosphorylation and the pERK/ERK ratio were significantly reduced in all the experiment groups compared with the controls. These results indicate that PFOS interfered with the ERK1/2 pathway in SH-SY5Y cells.

### 3.5. Effect of PFOS on the Relative Expression of miR-16 and miR-22

**Figure 5:**
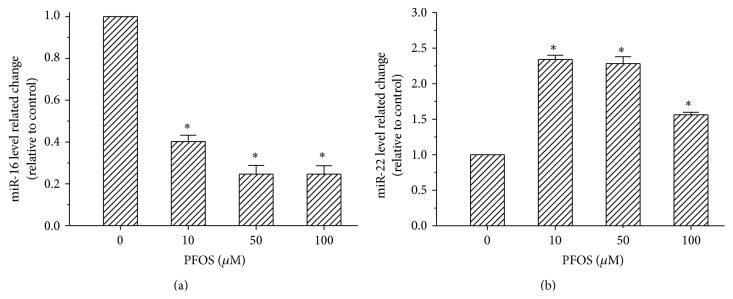
Effects of PFOS on the expression of miR-16 and miR-22 in SH-SY5Y cells. (a and b) miRNA (miR-16 and miR-22) expression was determined by quantitative real-time PCR, and the data were normalized to U6 as an internal control. Each data point was normalized to the control (DMSO), and the data are presented as mean ± SD from three independent experiments. ^*∗*^ *p* < 0.05, experimental values compared with control values.

BDNF mRNA has been previously reported to the target molecule of miR-16 [29, 30] and miR-22 [28]. To determine the relative mechanism through which PFOS alters BDNF expression, the relative expression of BDNF related miRNAs miR-16 and miR-22-3p was measured by RT-PCR. As shown in Figure 5, compared with the control, the relative expression of miR-16 was significantly reduced in all the experiment groups. However, compared with the control, the relative expression of miR-22 increased 234%, 228%, and 156% in the 10, 50, and 100 *μ*M dose groups, respectively, after a 48-hour treatment. These results indicate that PFOS influenced the relative expression of miR-16 and miR-22.

## 4. Discussion

PFOS is a ubiquitous, persistent pollutant in the environment and in living organisms. The health risks associated with PFOS exposure are concerning. One epidemiological study revealed that prenatal exposure to PFOS may affect neurodevelopment, especially gross-motor development, at 2 years of age [31]. The neurotoxicity of PFOS was previously associated with the calcium overload and abnormal increased level of calcium concentration in the hippocampus [14, 32]. The current study analysed the cytotoxicity of PFOS in human neuroblastoma SH-SY5Y cells, and the results indicated that PFOS exposure decreased cell viability and induced significant morphological abnormalities in SH-SY5Y cells. Additionally, PFOS exposure induced SH-SY5Y cell apoptosis in a dose-dependent manner. Previous studies demonstrated that PFOS induced apoptosis in SH-SY5Y cells through oxidative damage and an antioxidative deficit [33]. In the present study, we explored whether BDNF-ERK-CREB signalling was involved in the neurotoxicity induced by PFOS in SH-SY5Y cells.

The BDNF signalling molecule plays a critical role in neuronal development, synaptic plasticity, and long-term potentiation (LTP). BDNF regulates the synthesis of synaptic proteins and elevates synaptic efficacy, necessary precursors for appropriate neuronal function, survival, and apoptosis [22]. Impaired regulation of BDNF expression has been implicated in mood and anxiety disorders [34] and in cognitive and neurodegenerative diseases [35]; environment stimuli such as H_2_O_2_ reduced BDNF expression levels in SH-SY5Y cells [36]. As expected, in our study, BDNF mRNA and protein levels significantly decreased in a dose-dependent manner after a 48-hour exposure to various concentrations of PFOS. Such a decrease in BDNF would be detrimental to the propagation, maturation, survival, and synaptic function of SH-SY5Y cells. CREB also plays a central role in mediating the neurotrophin response and neuronal synaptic plasticity by promoting the transcription of neurotrophin-related genes, such as BDNF [24]. In the current study, CREB phosphorylation increased in a dose-dependent manner after PFOS exposure; however, CREB mRNA expression was not affected by PFOS. Although the level of pERK, an upstream activator of pCREB, was reduced in the present study, CREB can be activated by multiple signal pathways including PI3K/Akt and Ca/CaM/CaMK IV signalling [37, 38]. PFOS was demonstrated to activate the PI3K/Akt signaling pathway [39]. So we hypothesized that PFOS may increase the level of pCREB by PI3K/Akt and/or other pathways. Previous studies have demonstrated that PFOS induced Ca^2+^ influx in cultured neurons, subsequently elevating the levels of CaMKII*α* and pCREB [40, 41], which are both critical molecules downstream of calcium signalling that are important for neuronal cell structure and function. Zeng et al. found that increased pCREB expression may promote the transcription of c-fos, c-jun, IL-1*β*, and TNF-*α* [17], and these increases in transcription are associated with the neurodegeneration induced by neuroactive compounds, and they cause chronic glial activation and inflammation. In our study, the level of TrkB, an important membrane receptor for BDNF [21], was increased significantly compared with the control after a 48-hour incubation with 10 or 50 *μ*M PFOS; this may represent a compensatory response to decreased BDNF levels in SH-SY5Y cells following PFOS exposure. However, TrkB protein expression was significantly decreased compared with the control after a 48-hour exposure to 100 *μ*M PFOS, potentially due to a decompensated reaction indicating serious cytotoxicity in SH-SY5Y cells subjected to a high concentration of PFOS.

ERK is an important cell signalling molecule and a major member of the MAPK pathways. Research has revealed the potential of ERK signalling cascades to regulate diverse neuronal processes, such as cell death, differentiation, and synaptic plasticity [25]. Research by Lee et al. suggested that PFOS induced apoptosis of cerebellar granule cells by increasing pERK levels [42]. In the present study, ERK phosphorylation was reduced significantly in all the experimental groups compared with the controls, and the pERK/ERK ratio was significantly lower in all the experimental groups. The ERK pathway has a dual role in neuronal apoptosis [43], and the different effects of the ERK pathway may be due to the diverse types of analysed neurons, different stimuli, interplay with other MAPK pathways, and additional as yet unidentified factors. Therefore, downregulation of the pERK/ERK ratio may contribute to the PFOS-induced apoptosis of SH-SY5Y cells. Furthermore, because ERK is a downstream signalling molecule in the BDNF-TrkB signalling pathway, the decreased expression of BDNF may explain the decrease in the pERK/ERK ratio described herein.

Our previous studies demonstrated that prenatal exposure to PFOS induced an impairment of cognitive function associated with long-lasting changes in the expression of synapsins (synapsin 1 and synapsin 2) and synaptophysin and damage to the synaptic ultrastructure in rat hippocampi [44, 45]. Research by Wang et al. revealed an adverse effect of PFOS exposure on spatial learning and memory in rats that was associated with synaptic plasticity [20]. Liao et al. reported that a potential PFOS-induced enhancement of Ca^2+^ channels led to acute excitotoxic effects on synaptic function and chronically inhibited synaptogenesis in the brain [40], although the exact mechanism by which PFOS damaged synaptic function requires further investigation. Previous research demonstrated a BDNF-dependent increase in the levels of presynaptic synapsin 1 and synaptotagmin and an upregulation of synaptic protein expression, and activation of MAPK/ERK signalling was required for these effects [46, 47]. Thus, the expression changes in the BDNF-TrkB-CREB and ERK signalling pathways described herein may explain the changes in synaptic plasticity and synapsin protein expression in response to PFOS exposure.

However, the upregulated pCREB/CREB ratio did not increase BDNF transcript levels; thus, we further investigated the mechanism by which PFOS decreased BDNF expression in SH-SY5Y cells. The expression of BDNF is influenced by many factors, and epigenetic regulation plays an important role in BDNF expression changes induced by environment contaminants and in neurodegenerative diseases [48]. miRNAs are endogenous, small RNAs that bind to target mRNAs and regulate gene expression by repressing translation of target mRNAs. We examined the relative expression levels of miR-16 and miR-22 in SH-SY5Y cells after a 48-hour PFOS treatment as previous studies demonstrated that miR-16 [29] and miR-22 [28] regulated BDNF mRNA translation at the posttranscriptional level. Our results revealed that PFOS significantly increased the relative expression of miR-22, which may repress the translation of BDNF mRNA. However, the relative expression of miR-16, which directly targets the 3′UTR of BDNF mRNA and decreases BDNF protein levels, was decreased significantly after PFOS exposure. Besides miRNA, epigenetic modifications such as DNA methylation and histone modifications can affect BDNF expression in response to different environmental exposures [49, 50]. Our results indicate that PFOS most likely acts through miRNA-22 rather than miRNA-16 to suppress BDNF gene expression. The involvement of epigenetic modifications in PFOS' effects needs further investigation. This PFOS-induced decrease in miR-16 may cause a multitude of effects in SH-SY5Y cells, such as regulating the expression level of Tau protein, which plays a critical role in maintaining neuronal microtubule stability; furthermore, abnormal expression of Tau induces neuronal cytotoxicity and neurodegenerative diseases, such as Alzheimer's disease (AD) [51]. In addition, Johansson et al. found that neonatal exposure to PFOS (21 *μ*mol/kg body weight) significantly increased the expression of Tau protein in the mouse cerebral cortex [45].

## 5. Conclusion

The current study revealed that PFOS exposure induced the apoptosis of SH-SY5Y cells, decreased the expression of BDNF, and disturbed the BDNF-ERK-CREB signalling pathway. In addition, our results showed that the increase in miR-22 levels may contribute to the PFOS-induced decrease in BDNF expression. This work may contribute to the elucidation of a new mechanism of PFOS-induced neurotoxicity.

## Figures and Tables

**Table 1:** Primer sequences for quantitative real-time PCR.

Gene name	Primer	Product (bp)	Accession number
BDNF	Forward: AGCTGAGCGTGTGTGACAGTATTAG Reverse: ATTGCTTCAGTTGGCCTTTTGATAC	129	NM_170735

CREB	Forward: ACCTGCCATCACCACTGTAACG Reverse: GGGTGCTGTGCGAATCTGGGTA	289	NM_004379

ERK1	Forward: GCAGTTCTGGAATGGAAGGGTT Reverse: GGGGTTTGAATGAGATGAGGG	120	NM_001109891

ERK2	Forward: ATTACGACCCGAGTGACGAG Reverse: ACTGGGAAGAAGAACACCGA	200	NM_002745

*β*-Actin	Forward: TGAAGGTGACAGCAGTCGGTT Reverse: ACTTCCTGTAACAACGCATCTCATA	126	NM_001101
